# Self-reported low self-esteem due to poor maternal care improves with the existential approach in bipolar disorder: a case report

**DOI:** 10.3389/fpsyt.2023.1243188

**Published:** 2023-08-29

**Authors:** Hirofumi Hirakawa, Takeshi Terao, Nobuko Kawano

**Affiliations:** ^1^Department of Neuropsychiatry, Oita University Faculty of Medicine, Yufu, Japan; ^2^Usa Hospital, Oita, Japan; ^3^Department of Psychology, Faculty of Welfare and Health Sciences, Oita University, Oita, Japan

**Keywords:** existential approach, bipolar disorder, self-esteem, maternal care, awakening experience

## Abstract

Parental nurturing attitudes influence children and are linked to the establishment of self-esteem. Females who have experienced poor maternal care during their childhood may have low self-esteem, and this factor may significantly augment the likelihood of depression. Particularly, childhood maltreatment among individuals with bipolar disorder is associated with unfavorable clinical features, such as a heightened risk of severe manic, depressive, or psychotic symptoms, as well as suicide attempts. Here, we report a case of a woman with bipolar disorder who had self-reported low self-esteem due to poor maternal care, which subsequently improved *via* an existential approach. This existential approach confers meaning to the lives of every individual, even in the face of adversity. Our findings suggest that the existential approach may enable the discovery of more positive life values during times of hardship and could improve self-reported low self-esteem due to poor maternal and change the way of life in patients with bipolar disorder.

## Background

Bipolar disorder is a chronic disorder characterized by fluctuations in mood state and energy as well as recurrent episodes of elevated mood and depression (1). Patients with bipolar disorder present with difficulties in interpersonal relationships, education, or employment (2, 3). Individuals diagnosed with bipolar disorder, the etiology of which remains obscure, may feel despair at the absurdity of what has happened to them, thereby compromising their outlook towards the future and diminishing their overall quality of life (QoL). Indeed, individuals with bipolar disorder exhibit reduced QoL compared with healthy adults, even in a euthymic state, wherein mood symptoms remain stable (4, 5). Low self-esteem in patients with psychiatric disorders is related to QoL (6). Particularly, bipolar disorder with low self-esteem is likely to exert a more deleterious impact on the QoL.

Child-parent relationships are essential in an individual’s psychological development. Parental nurturing attitudes influence children and are linked to the establishment of self-esteem. In females, experiencing poor maternal care during their childhood may play a part in the emergence of low self-esteem, and this factor may significantly augment the likelihood of depression (7, 8). Particularly, childhood maltreatment among individuals with bipolar disorder is associated with unfavorable clinical features, such as a heightened risk of severe manic, depressive, or psychotic symptoms, as well as suicide attempts (9).

The existential approach confers meaning to the lives of every individual, even in the face of adversity. For individuals with bipolar disorder, especially those with low self-esteem, not only psychotropic treatment to stabilize mood symptoms but also the existential approach proves particularly efficacious and enables the discovery of positive life values during times of hardship. Here, we report a case of a woman with bipolar disorder who had self-reported low self-esteem due to poor maternal care, which subsequently improved *via* an existential approach.

## Case presentation

A 39 years-old Japanese woman with bipolar II disorder regularly visited our hospital. During her formative years, she resided alongside her parents and younger sister. However, she had no recollection of maternal affection from her childhood, which resulted in her loneliness and isolation. After she graduated from the university department of social welfare, she began working at a welfare institution and married a co-worker at the age of 27. At the age of 28, she gave birth to her first child. She was tired of housework and childcare and felt depressed. After returned to work, her depression worsened. She consulted a psychiatrist and was diagnosed with depression. Subsequently, she resigned from her employment, and her depression improved with antidepressants. When she was 33 years old, she exhibited a hypomanic state and was diagnosed with bipolar II disorder. Thereafter, she was hospitalized twice for depressive episodes. At the age of 36, she was transferred to our hospital, and her psychotropics changed. She was treated with 200 mg per day of lamotrigine, 40 mg per day of lurasidone, 50 mg per day of quetiapine, 2 mg per day of bromazepam, and 5 mg per day of zolpidem, and she was in a euthymic state. At the age of 37, her depressive symptoms worsened when her husband requested a divorce, although they improved with weekly supportive psychotherapy without psychotropic alterations. Eventually, her husband withdrew from the divorce. Although she was in a euthymic state for the next year and a half, her self-reported low self-esteem left her uncertain about how to navigate her future. The guidelines of International Society for Bipolar Disorders stated that adjunctive psychosocial interventions may be useful for in maintenance treatment to prevent relapse (10). The psychosocial interventions include psychoeducation, cognitive behavioral therapy, family-focused therapy, interpersonal and social-rhythm therapy, and peer support. She was in a euthymic state and under remission, grappling with profoundly existential problem regarding her life moving forward. We concurred with the proven efficacy of forementioned psychosocial interventions in the management of bipolar disorder, they remained insufficient in addressing the profound existential problem of life. Therefore, we recommend the introduction of an existential approach. In the existential approach, we performed a life scan which consisted of four sessions (11). In session 1 (S1), the patient recalled past successful (reasonable) experiences. In session 2 (S2), the patient recalled past miserable (unreasonable) experiences. In session 3 (S3), the patient thought about the purpose and meaning of life not attributed to common sense, but to her proper thoughts, accepting herself as she was and understanding that she was happy when she thought so. In session 4 (S4), the patient confirmed that an individual would live better in an uncertain future. In each session, she was prompted to perform a life scan of the session’s theme and subsequently document her experiential content or thoughts. Thereafter, according to her note, a 30 min interview was conducted with a psychiatrist.

Before starting the existential approach, as she was in a euthymic state, the Hamilton rating scale for depression score was 6 points, the Beck depression inventory score was 13 points, and the Young mania rating scale score was 0. The scores on the parental bonding instrument (PBI), a brief self-report measure that relies on the participant’s retrospective judgment of the quality of his or her relationship with each parent, were 13 points for paternal care, 24 points for paternal overprotection, 8 points for maternal care, and 17 points for maternal overprotection, revealing that she experienced remarkably poor maternal care. Her mental condition was assessed at baseline and after each session using the self-compassion scale (SCS) and the purpose in life test (PIL). Her quality of life was assessed at baseline and after the existential approach using the World Health Organization Quality of Life-26 (WHOQOL-26).

**S1:** successful experiences, “When I was a young child, I discovered the sheer delight of drawing; it brought me great joy to receive compliments from my friends for my artistic prowess. Drawing has become a daily comfort for me, as I have created new illustrations and shared them on social media. It is truly gratifying to receive likes and comments on my drawings. During my college years, I met impressive people. Even today, I am in contact with my friends. Previously, I approached my work sincerely. I had the pleasure of dating a colleague whom I respected greatly and eventually became hitched with. Presently, my greatest joy lies in watching my adorable children. Given my strained relationship with my mother, I am determined not to put my children through the same pain and suffering that I have experienced.” The therapist commended her successful (reasonable) experience.

**S2:** miserable experiences, “I cannot remember my mother ever showing me any affection, and I do not have any recollection of seeing her smile while I was growing up. There were constant feelings of sadness and hopelessness. In my youth, I often felt lonely, but I accepted this as my reality. I was accepted into the psychology program at a university, but due to financial problems, I had to enroll in a social welfare program at a different university. To be honest, I really wanted to attend art school, but I never felt like I was allowed to do what I wanted to do in my life. After having a child, I pushed myself to do a better job of taking care of my child, but each day, it was a struggle. When I returned to work, I was overwhelmed by balancing work and caring for my child, leading me to push myself too hard. I felt that no matter what I did, I could not succeed and thus felt worthless. Eventually, I fell ill, took a leave of absence from work, and finally resigned. Two years ago, my husband suddenly filed for divorce, which completely blindsided me and turned my world upside down. Although the divorce was later annulled, relations between me and my husband have been strained ever since.” Her patience and perseverance in miserable (unreasonable) experiences were respected by the therapist.

**S3:** the purpose and meaning of life, “I have been through a lot of hard times, but I think I have done well. Currently, I can view my life from a positive perspective. My children bring me joy and affection, which are unshakeable sentiments. However, I am afraid that my children will become the whole meaning of my existence. I look back on many things from my past and want to live more of my own life. I want to face my life properly. I like drawing. I have loved it since I was a child, and I really want to pursue higher education in the arts. I want to continue drawing on my life. From an early age, I have harbored a belief that “happiness always ends” and “good things inevitably crumble.” Despite the happiness I presently experience with my children in my daily life, I have always harbored the belief that my good fortune will eventually dissipate. However, upon reflection on my accomplishments, I have come to realize that positive outcomes are not always transient.”

**S4:** to live better in the uncertain future, “I want to maintain my unwavering love for my children and ensure that they understand their significance to me. I aspire for them to have the freedom to grow and explore spontaneously without any inhibition or limitations. It is essential that I prioritize my own well-being and indulge in activities that bring me joy. I aspire to be compassionate and considerate towards both myself and others. I aspire to discover joy in the little things that comprise my everyday life, treat myself with compassion, and relish my experience of living to the fullest. Drawing serves as one of my preferred forms of self-expression, and I intend to pursue it throughout my life. In addition, I am interested in taking on work.”

**Figure 1:**
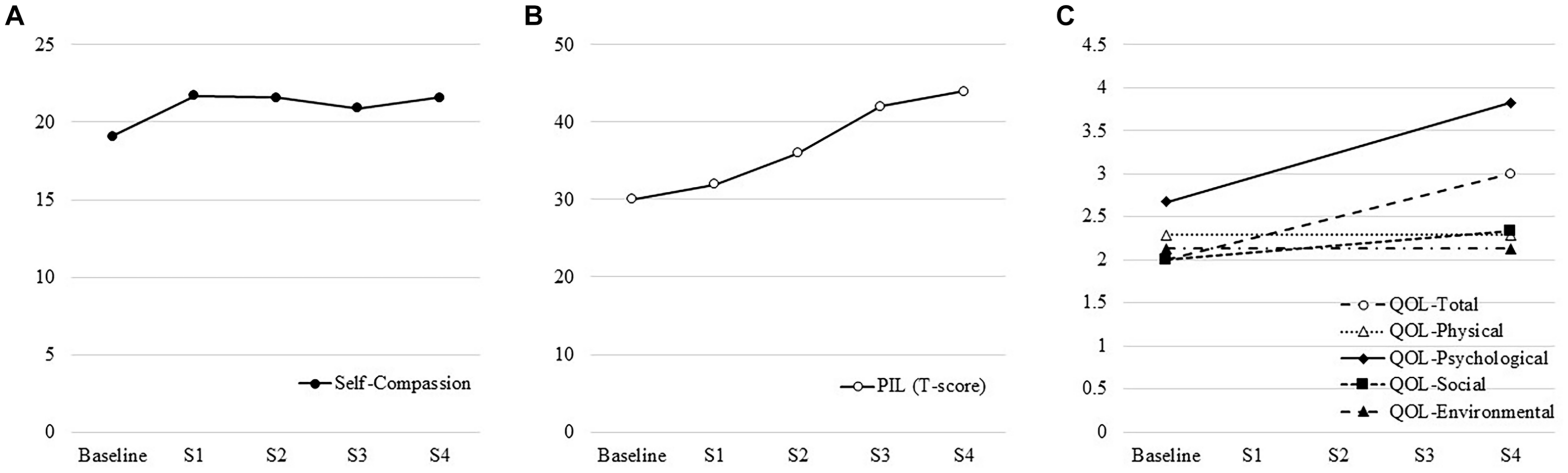
**(A)** Time-course of the self-compassion scale. Her self-compassion scale increased through existential approach. **(B)** Time-course of the purpose in life test. Her general sense of meaning and purpose in life increased through existential approach. **(C)** Time-course of World Health Organization Quality of Life-26. Her quality of life increased through existential approach.

Through the existential approach, her self-reported low self-esteem improved, and she began to view her life positively. Figures 1A,B shows the time courses of the SCS and PIL scores. Her self-compassion and general sense of meaning and purpose improved. In addition, her quality of life, assessed by WHOQOL-26, increased, especially in terms of psychological factors (Figure 1C).

After completing the existential approach, she was still in the euthymic state. A month later, she started new work as a cartoon illustrator, which she could do at home. Previously, she worked at a welfare institution, and this was her first time working on drawings. Reflecting on this new chapter in her life, she said, “I was pleasantly surprised and overjoyed to have the opportunity for a job in my favorite field. I will do my best to do this job. However, I would like to continue drawing as a hobby.”

## Discussion

In this case, she had self-reported low self-esteem due to poor maternal care, and she felt that she was never allowed to do what she wanted to do in her life. The study by Parker (12) using PBI found that persons with maternal care scores of 10 or less were at special risk for depression; when the cut-off point for maternal care was set at 10, the unadjusted odds ratios rose dramatically to 14.9, and when poor maternal care was considered along with other potential childhood risk factors, the special risk for depression was found to have the largest odds ratio. In our case, the score of maternal care on the PBI was 8 points, which was less than the cut-off points of the previous study, indicating that she experienced remarkably poor maternal care. In the past, she was unable to receive adequate nurturing from her mother, and her absurdity could not change. For such patients, the existential approach could be effective in confronting and searching for the meaning of one’s existence in a life full of hardship.

In the existential approach, the therapist helps a hopeless individual realize that as a “person being-in-time,” he or she is always living in limitless temporality where change is inevitable and the future bestows hope based on Heidegger’s philosophy (13). The therapist assists distraught individuals in discovering meaningfulness, finding purpose, and actualizing self-love (or self-esteem) by facilitating deeper self-awareness, appreciation, and understanding of who they are, who they have been, and their unique process of becoming. Our previous study showed that the combination of existential and mindfulness-based interventions (EXMIND) increased the scores of self-compassion in an apparently healthy participant (11). Furthermore, a study to identify the predictors of response patterns to EXMIND revealed that maternal overprotection might predict consistent improvement in self-compassion (14). The experience of maternal overprotection in childhood may lead to decreased self-compassion, while EXMIND could help to increase self-compassion (14). The existential approach supports the uniqueness of each individual and helps find meaning in one’s life with maternal overprotection in childhood (14). Nonetheless, the average score for maternal care exceeded 20 points in this study, and the participants did not exhibit any deficiencies in maternal nurturing, which may impede the determination of the effectiveness of the existential approach in poor maternal care (14). The present case was characterized by markedly poor maternal care; however, with the adoption of an existential approach, self-compassion improved. Therefore, the existential approach may also effective in cases of poor maternal care. Through the existential approach, she was able to develop a positive outlook toward life despite the experience of poor maternal care during her childhood, which resulted in improvements in her self-reported self-esteem and self-kindness and increases in the scores of QoL and PIL.

In the session of S4, the final session of the existential approach, she talked about her children first. Considering her experience of poor maternal care, it is plausible that she might have been projecting her childhood self onto her own children, seeking to fulfill the love and nurturing that she lacked from her mother. However, her own statement, “I aspire for them to have the freedom to grow and explore spontaneously without any inhibitions or limitations,” reveals her high regard for her children’s self-determination and self-sufficiency; the statement is a forward-looking statement that envisages their future independence and autonomy. Furthermore, as a result of this existential approach, she developed an awareness of life’s finite nature, allowing her to prioritize and make decisions about what was truly important in her life. This “awakening experience,” a concept initiated by Yalom (15), might have led her to recognize that her love for her children is paramount. It is essential to note that every mother-child relationship is distinct and deeply personal, and for her, the bond with her child is viewed as an existential component of her being. She recalled a strained relationship with her mother and was determined not to put her children in the same pain and suffering. She expressed her desire to be loving and have a positive influence on her children. This could be Yalom’s concept of “rippling,” which suggests that our actions create a series of influences that spread like ripples in a pond, affecting the world around us (15).

Drawing emerged as a prominent theme of her life. In sessions of S1 (successful experiences) and S3 (the purpose and meaning of life), she expressed her love for drawing. She also mentioned that she was afraid that her children would become the entire meaning of her existence. In the session of the future, she resolved to continue drawing, which is a favorable mode of self-expression. Surprisingly, she started a new work as a cartoon illustrator after completing the existential approach. While an increase in her self-reported self-esteem might have contributed to this, we contend that it was more likely a result of the positive impact of the existential approach. Insights into life’s finite nature allowed her to make choices in line with the experiences that were most important in her life. Through works of art, creativity, and play, we transform our experience of the world, find new meaning, and engage in activities or work that are valuable and point to something larger than ourselves; we, thus, create meaning in our lives (16).

It might be more robust evaluation of self-esteem through the utilization of a quantifiable questionnaire, such as the Rosenberg self-esteem scale (17). Nevertheless, we did not assess self-esteem such questionnaire in this case, we could not demonstrate improvement by scoring the self-esteem. In addition, this is only a single-case report and the evidence level was low. Further study is needed to investigate the efficacy of existential approach for bipolar disorders.

In conclusion, our findings suggest that the existential approach can improve self-reported low self-esteem due to poor maternal care and change the way of life in patients with bipolar disorder.

## Data availability statement

The raw data supporting the conclusions of this article will be made available by the authors, without undue reservation.

## Ethics statement

Written informed consent was obtained from the individual(s) for the publication of any potentially identifiable images or data included in this article.

## Author contributions

HH is a primary doctor of the patients and wrote the manuscript. TT and NK provided constructive criticism it. All authors contributed to the article and approved the submitted version.

## Conflict of interest

The authors declare that the research was conducted in the absence of any commercial or financial relationships that could be construed as a potential conflict of interest.

## Publisher’s note

All claims expressed in this article are solely those of the authors and do not necessarily represent those of their affiliated organizations, or those of the publisher, the editors and the reviewers. Any product that may be evaluated in this article, or claim that may be made by its manufacturer, is not guaranteed or endorsed by the publisher.
